# Early diagnosis of sinusoidal obstruction syndrome after hematopoietic stem cell transplantation, with modified diagnostic criteria including refractory thrombocytopenia

**DOI:** 10.1002/jha2.728

**Published:** 2023-06-01

**Authors:** Hiroya Ichikawa, Kimikazu Yakushijin, Yoshiharu Miyata, Hirofumi Kanehira, Miki Joyce, Yuri Hirakawa, Sakuya Matsumoto, Shigeki Nagao, Rina Sakai, Keiji Kurata, Akihito Kitao, Yasuyuki Saito, Shinichiro Kawamoto, Katsuya Yamamoto, Mitsuhiro Ito, Tohru Murayama, Hiroshi Matsuoka, Hironobu Minami

**Affiliations:** ^1^ Division of Medical Oncology/Hematology Department of Medicine Kobe University Hospital and Graduate School of Medicine Kobe Japan; ^2^ BioResource Center Kobe University Hospital Kobe Japan; ^3^ Jerome Lipper Multiple Myeloma Center Department of Medical Oncology Dana‐Farber Cancer Institute Harvard Medical School Boston Massachusetts USA; ^4^ Division of Molecular and Cellular Signaling Kobe University Graduate School of Medicine Kobe Japan; ^5^ Transfusion Medicine and Cell Therapy Kobe University Hospital and Graduate School of Medicine Kobe Japan; ^6^ Laboratory of Hematology Division of Medical Biophysics Kobe University Graduate School of Health Sciences Kobe Japan; ^7^ Department of Hematology Hyogo Cancer Center Akashi Japan; ^8^ Cancer Center Kobe University Hospital Kobe Japan

**Keywords:** diagnostic criteria, hematopoietic stem cell transplantation, sinusoidal obstruction syndrome

## Abstract

Sinusoidal obstruction syndrome (SOS) is a fatal complication of hematopoietic stem cell transplantation (HSCT). Early diagnosis for SOS can improve clinical outcomes significantly. Here, we performed a retrospective study to investigate the Cairo diagnostic criteria, in which SOS was defined as the development of two or more in seven events, including transfusion‐refractory thrombocytopenia. Among 154 cases of allogeneic HSCT, 10 cases of SOS using the European Society for Blood and Marrow Transplantation criteria (EBMT16) as the reference standard were identified. The original Cairo criteria could diagnose SOS 5 days earlier than any other established criteria, with some false‐positive results (sensitivity = 100.0%; specificity = 72.2%). When the cutoff was set to three events for the Cairo criteria, the diagnosis of SOS could be made 3 days earlier than that using the EBMT16 criteria, with comparable precision (specificity = 86.1%). The accuracy of the Cairo criteria improved further when the cutoff point was set to four (specificity = 93.8%). The fulfillment of the Cairo criteria was associated with high mortality. Based on our results, the Cairo criteria were also considered clinically useful, especially at three or four cutoff points. Further studies are required to validate and refine the criteria.

## 1 INTRODUCTION

Allogeneic hematopoietic stem cell transplantation (allo‐HSCT) is widely administered as a curative treatment for various hematological diseases. However, allo‐HSCT can cause potentially fatal complications, such as graft‐versus‐host disease and sinusoidal obstruction syndrome (SOS), previously known as a veno‐occlusive disease. The incidence of SOS has been estimated at approximately 10%–15% after allo‐HSCT [1, 2, 3]. Severe SOS with multiple organ failure (MOF) is associated with an extremely high mortality rate (> 80%) [3]. Some studies have demonstrated that early initiation of treatment for SOS is related to significant improvement in clinical outcomes [4, 5, 6]. Therefore, early diagnosis is necessary to treat SOS effectively.

Owing to the difficulty in definitive diagnoses by invasive procedures, such as liver biopsy immediately after HSCT, SOS had been diagnosed with the modified Seattle criteria and/or the Baltimore criteria as surrogate gold standards for a long time [7, 8]. The Baltimore criteria contain the mandatory event of hyperbilirubinemia (≥ 2 mg/dL) for diagnosis of SOS. As the elevation of bilirubin can be a delayed manifestation in SOS [9], this event potentially leads to a delay in the diagnosis. In 2016, the European Society for Blood and Marrow Transplantation (EBMT) advocated the revised diagnostic criteria for SOS in adults (EBMT16 criteria) [9]. Using the EBMT16 criteria, “classical SOS” is diagnosed with almost identical events to the Baltimore criteria, including hyperbilirubinemia. Therefore, these criteria could also lead to a delay in diagnosis.

Recently, Cairo et al. have proposed their own set of original diagnostic criteria [10]. They defined SOS as the development of two or more of the seven events (Table 1). The unique characteristics of their criteria are as follows: refractory thrombocytopenia and Doppler ultrasound findings. Furthermore, hyperbilirubinemia is not a mandatory event, and the threshold for hyperbilirubinemia is lower than those in other established criteria. While the “Cairo criteria” are expected to allow for earlier diagnosis compared to the other established criteria, no verification and validation studies have been performed yet. In this study, we verified the “Cairo criteria” by performing a retrospective investigation of patients who underwent allo‐HSCT.

**TABLE 1:** Diagnostic criteria for SOS.

Established criteria			Modified diagnostic criteria	
Modified Seattle criteria [7]	Baltimore criteria [8]	EBMT16 criteria (classical SOS) [9]	Cairo criteria [10]	
The presence of two or more of the following within 20 days	The presence of hyperbilirubinemia: ≥ 2 mg/dL and two or more of the following within 21 days		The presence of two or more of the following	Or the presence of either of the following
(1) Hyperbilirubinemia: > 2 mg/dL (2) Hepatomegaly and/or Right upper quadrant pain (3) Weight gain: > 2%	(1) Hepatomegaly (2) Ascites (3) Weight gain: ≥ 5%	(1) Painful hepatomegaly (2) Ascites (3) Weight gain: > 5%	(1) Hyperbilirubinemia: > upper institutional limits (2) Weight gain: ≥ 5% (3) Refractory thrombocytopenia (4) Hepatomegaly (5) Right upper quadrant pain (6) Ascites (7) Reversal of portal venous flow	(1) Liver biopsy finding (2) Elevated portal venous wedge pressure

Abbreviations: EBMT, European Society for Blood and Marrow Transplantation; SOS, sinusoidal obstruction syndrome.

## 2 METHODS

### 2.1 Patients

Data from patients aged ≥ 18 years who underwent allo‐HSCT at Kobe University Hospital between January 2012 and July 2022 were retrospectively analyzed. This study was approved by the Ethics Committee of Kobe University Hospital (No. B220141), and the requirement for informed consent was waived due to the retrospective nature of the study. The patients who participated in this study were offered the opportunity to opt‐out. This study was conducted in accordance with the principles of the Declaration of Helsinki.

### 2.2 Definitions of SOS and related terms

The modified Seattle criteria define SOS as the development of two or more of the following events within 20 days after HSCT: (1) hyperbilirubinemia (> 2 mg/dL), (2) hepatomegaly and/or right upper quadrant pain, and (3) weight gain (> 2% compared to baseline weight pre‐HSCT) [7]. The Baltimore criteria define SOS as the development of hyperbilirubinemia (≥ 2 mg/dL) and two or more of the following events within 21 days after HSCT: (1) hepatomegaly, (2) ascites, and (3) weight gain (≥ 5% compared to baseline weight pre‐HSCT) [8]. The EBMT16 criteria define “classical SOS” as the development of hyperbilirubinemia (≥ 2 mg/dL) and two or more of the following events within 21 days after HSCT: (1) painful hepatomegaly, (2) ascites, and (3) weight gain (> 5% compared to baseline weight pre‐HSCT) [9]. We defined “classical SOS” using the EBMT16 criteria as the reference standard.

We configured SOS diagnosis by the “Cairo criteria” as the development of two or more of the following events within 21 days after HSCT: (1) hyperbilirubinemia beyond the upper institutional limits (1.5 mg/dL in our institution), (2) weight gain (≥ 5% compared to baseline weight pre‐HSCT), (3) platelet transfusions consistent with refractory thrombocytopenia (24 h corrected count increment falling below 4.5 × 10^9^/L) [11], (4) hepatomegaly, (5) right upper quadrant pain, (6) ascites, and (7) reversal of portal venous flow (hepatofugal flow) detected on Doppler ultrasound of the liver [10]. Either of the following events would also be defined as SOS: a liver biopsy consistent with SOS or elevated portal venous wedge pressure by direct measurements.

MOF was defined as coexistence with renal failure (serum creatinine ≥ 3 × the upper limit of normal or dependence of dialysis) and/or respiratory failure (oxygen saturation < 90% on room air, requirement for oxygen supplementation, or ventilator dependence) [1]. Remission of SOS was defined as the resolution of all signs and symptoms of established SOS diagnostic criteria [1]. The severity of SOS for cases who fulfilled any of the established criteria was graded using the EBMT16 grading for adults [9]. Conditioning regimens were classified as myeloablative conditioning if any of the following regimens were used: total body irradiation > 8 Gy, intravenous busulfan ≥ 7.2 mg/kg, or melphalan ≥ 140 mg/m^2^. Other conditioning regimens were classified as reduced‐intensity conditioning [12].

### 2.3 Statistical analysis

Categorical and continuous variables of case characteristics were compared using Fisher's exact test and the Mann–Whitney U test, respectively. The intervals of diagnosed days between each criterion and the EBMT16 criteria were compared using the Wilcoxon signed‐rank sum test. To compare the specificity of each criterion, the negative proportion of each test in non‐SOS cases was compared using the McNemar test with Yates's continuity correction [13]. The incidence of the SOS estimated by each criterion was calculated using the method of cumulative incidence, considering non‐SOS mortality as the competing risk. Using the receiver operating characteristic (ROC) curve, the optimal cutoff was determined as the point closest to the upper left corner. Overall survival was estimated using the Kaplan–Meier method with the log‐rank test. Statistical significance was defined as a two‐tailed *p* value < 0.05. All statistical analyses were performed using R version 4.1.2 and EZR version 1.55 [14].

## 3 RESULTS

### 3.1 Case characteristics

Data from 135 patients (154 transplant cases) were analyzed. The median follow‐up period for survivors was 1008 days (33–3823) after allo‐HSCT. The characteristics of all the cases are shown in Table 2. The number of cases who developed “classical SOS” according to the EBMT16 criteria was 10 (6.5%). Grade 3 and 4 SOS was observed in two and eight cases, respectively, and no grade 1 or 2 SOS was observed. In our cohort, haploidentical‐related transplantation was not performed. Ursodeoxycholic acid for SOS prophylaxis was routinely used in our hospital during the physicians estimated it to be required. A summary of the treatment and clinical outcomes for the cases with classical SOS is shown in Table S1.

**TABLE 2:** Case characteristics.

		Total cases	Classical SOS	No SOS	
*N*		154	10	144	*p* value
Median (range) age, years		52 (19–69)	47 (28–57)	52 (19–69)	0.23
Sex (%)	Male	91 (59.1)	5 (50.0)	86 (59.7)	0.79
	Female	63 (40.9)	5 (50.0)	58 (40.3)	
Disease (%)	AML	59 (38.3)	3 (30.0)	56 (38.9)	0.57
	ALL/LBL	32 (20.8)	1 (10.0)	31 (21.5)	
	MDS	16 (10.4)	1 (10.0)	15 (10.4)	
	CML/MPN	10 (6.5)	2 (20.0)	8 (5.6)	
	ML	15 (9.7)	2 (20.0)	13 (9.0)	
	MM	4 (2.6)	0 (0.0)	4 (2.8)	
	ATL	8 (5.2)	0 (0.0)	8 (5.6)	
	AA	3 (1.9)	0 (0.0)	3 (2.1)	
	Other^a^	7 (4.5)	1 (10.0)	6 (4.2)	
Disease status (%)	CR	95 (61.7)	1 (10.0)	94 (65.3)	<0.01
	PR	13 (8.4)	1 (10.0)	12 (8.3)	
	SD	2 (1.3)	0 (0.0)	2 (1.4)	
	PD	35 (22.7)	7 (70.0)	28 (19.4)	
	Unevaluable	9 (5.8)	1 (10.0)	8 (5.6)	
ECOG PS (%)	0	32 (20.8)	0 (0.0)	32 (22.2)	<0.01
	1	106 (68.8)	5 (50.0)	101 (70.1)	
	2	10 (6.5)	2 (20.0)	8 (5.6)	
	3	4 (2.6)	3 (30.0)	1 (0.7)	
	4	2 (1.3)	0 (0.0)	2 (1.4)	
HCT‐CI (%)	0	40 (26.0)	0 (0.0)	40 (27.8)	0.11
	1–2	34 (22.1)	4 (40.0)	30 (20.8)	
	≥3	80 (51.9)	6 (60.0)	74 (51.4)	
Source (%)	BM	63 (40.9)	2 (20.0)	61 (42.4)	0.14
	PBSC	28 (18.2)	4 (40.0)	24 (16.7)	
	CB	63 (40.9)	4 (40.0)	59 (41.0)	
Donor (%)	Related	30 (19.5)	3 (30.0)	27 (18.8)	0.65
	Unrelated	124 (80.5)	7 (70.0)	117 (81.2)	
Number of transplantation (%)	1	130 (84.4)	9 (90.0)	121 (84.0)	0.85
	2	22 (14.3)	1 (10.0)	21 (14.6)	
	3	2 (1.3)	0 (0.0)	2 (1.4)	
HLA serotype mismatch (%)	No	73 (47.4)	5 (50.0)	68 (47.2)	1.00
	Yes	81 (52.6)	5 (50.0)	76 (52.8)	
HLA genotype mismatch (%)	No	64 (41.6)	5 (50.0)	59 (41.0)	0.82
	Yes	90 (58.4)	5 (50.0)	85 (59.0)	
Conditioning (%)	MAC	66 (42.9)	5 (50.0)	61 (42.4)	0.89
	RIC	88 (57.1)	5 (50.0)	83 (57.6)	
BU containing regimen (%)		50 (32.5)	3 (30.0)	47 (32.6)	1.00
TBI containing regimen (%)		134 (87.0)	8 (80.0)	126 (87.5)	0.84
GVHD prophylaxis (%)	TAC + MMF	126 (81.8)	8 (80.0)	118 (81.9)	0.96
	CyA + MMF	17 (11.0)	1 (10.0)	16 (11.1)	
	TAC alone	10 (6.5)	1 (10.0)	9 (6.2)	
	Other	1 (0.6)	0 (0.0)	1 (0.7)	
Previous exposure to GO (%)		1 (0.6)	0 (0.0)	1 (0.7)	1.00
Previous exposure to INO (%)		2 (1.3)	0 (0.0)	2 (1.4)	1.00
UDCA for SOS prophylaxis (%)	Yes	148 (96.1)	10 (100.0)	138 (95.8)	0.39
	No	6 (3.9)	0 (0.0)	6 (4.2)	

Abbreviations: AA, aplastic anemia; ALL/LBL, acute lymphoblastic leukemia/lymphoma; AML, acute myeloid leukemia; ATL, adult T‐cell leukemia/lymphoma; BM, bone marrow; BU, busulfan; CB, cord blood; CML/MPN, chronic myeloid leukemia/myeloproliferative neoplasm; CR, complete remission; CyA, cyclosporine; ECOG PS, Eastern Cooperative Oncology Group performance status; GO, gemtuzumab ozogamicin; GVHD, graft‐versus‐host disease; HCT‐CI, hematopoietic cell transplant comorbidity index; HLA, human leukocyte antigen; INO, inotuzumab ozogamicin; MAC, myeloablative conditioning; MDS, myelodysplastic syndrome; ML, malignant lymphoma; MM, multiple myeloma; MMF, mycophenolate mofetil; PBSC, peripheral blood stem cell; PD, progressive disease; PR, partial remission; RIC, reduced‐intensity conditioning; SD, stable disease; SOS, sinusoidal obstruction syndrome; TAC, tacrolimus; TBI, total body irradiation; UDCA, ursodeoxycholic acid.

^a^The “other” disease category contains T‐cell prolymphocytic leukemia, chronic active Epstein–Barr virus infection, and so on.

### 3.2 Evaluation of established diagnostic criteria and the Cairo diagnostic criteria

**FIGURE 1:**
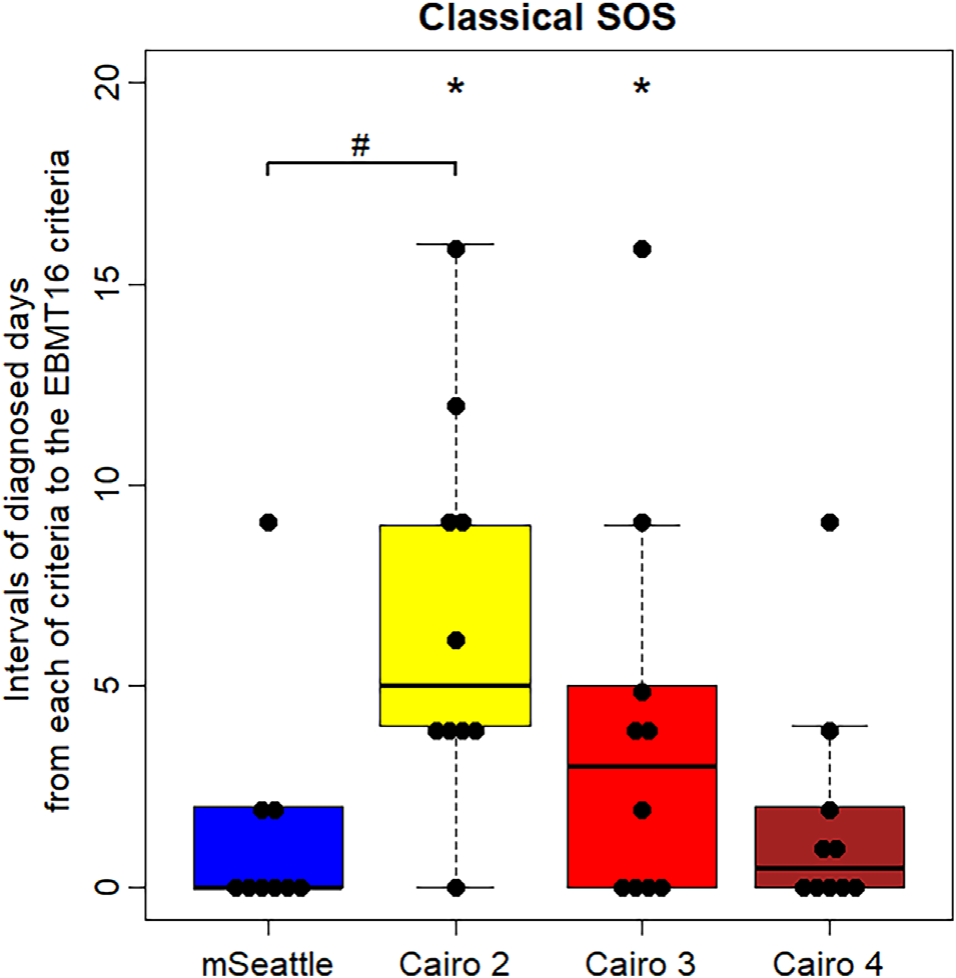
Intervals of diagnosed days from each of the criteria groups to the EBMT16 criteria. By applying each criterion, all cases could be diagnosed as soon as, or earlier than the EBMT16 criteria. *p < 0.05. # Original Cairo criteria could diagnose SOS significantly earlier than the modified Seattle criteria (*p* < 0.05). Abbreviations: Cairo 2, original Cairo criteria with two or more events; Cairo 3, Cairo criteria with three or more events; Cairo 4, Cairo criteria with four or more events; EBMT, European Society for Blood and Marrow Transplantation; mSeattle, modified Seattle criteria.

**FIGURE 2:**
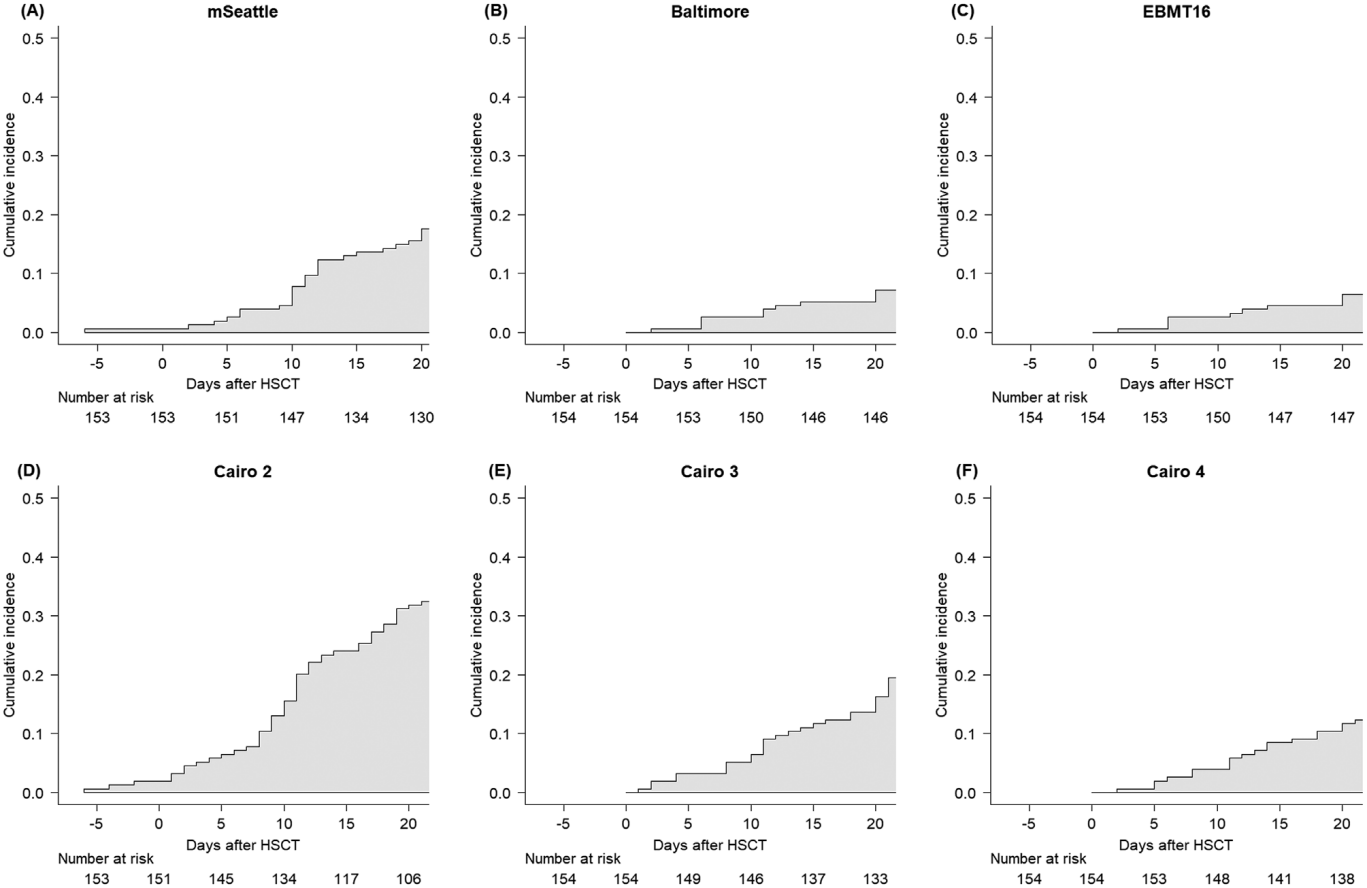
The cumulative incidence of SOS estimated by each of the criteria groups. Deaths without SOS as a competing risk were not observed within 21 days after allo‐HSCT. Abbreviations: Cairo 2, original Cairo criteria with two or more events; Cairo 3, Cairo criteria with three or more events; Cairo 4, Cairo criteria with four or more events; EBMT, European Society for Blood and Marrow Transplantation; HSCT, hematopoietic stem cell transplantation; mSeattle, modified Seattle criteria.

The median numbers of days on which SOS was diagnosed using each criterion are shown in Table S2. Among the 10 cases with “classical SOS,” three were diagnosed earlier with the modified Seattle criteria than with the EBMT16 criteria (Figure 1). Differences in the time of diagnosis between the modified Seattle criteria and the EBMT16 criteria were not statistically significant (median, 0.0 days; range, 0–9 days; *p* = 0.174). No differences were observed in the diagnosis time between the Baltimore and EBMT16 criteria. Among the 10 cases with classical SOS, nine were diagnosed significantly earlier with the original Cairo criteria than with the EBMT16 criteria (median, 5.0 days; range, 0–16 days; *p* = 0.008). Furthermore, seven cases were diagnosed significantly earlier with the original Cairo criteria than with the modified Seattle criteria (median, 4.0 days; range, 0–12 days; *p* = 0.022). The cumulative incidence of SOS diagnosed according to each criterion is shown in Figure 2. Non‐SOS death as a competing risk was not observed within 21 days after allo‐HSCT.

**FIGURE 3:**
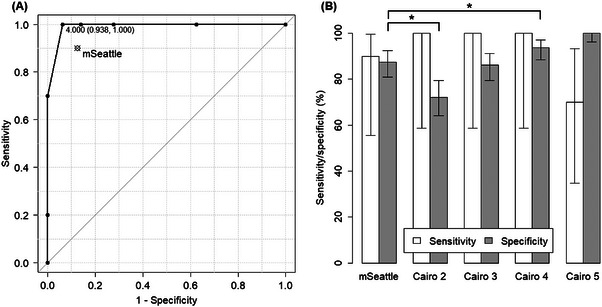
Sensitivity and specificity of the Cairo criteria. (A) ROC curve of the Cairo criteria. The EBMT16 criteria were used as a reference standard. The sensitivity/specificity of the modified Seattle criteria is pointed out. Optimal cutoff point is identified as four. (B) Details of sensitivity/specificity for each cutoff point of the Cairo criteria. 95% confidence intervals for each parameter are shown. *p < 0.05. Abbreviations: EBMT, European Society for Blood and Marrow Transplantation; mSeattle, modified Seattle criteria; ROC curve, receiver operating characteristic curve.

Considering “classical SOS” in the EBMT16 criteria as the reference standard of SOS, the sensitivity of the modified Seattle, Baltimore, and original Cairo criteria was 90.0%, 100.0%, and 100.0%, respectively (Figure 3 and Table S3). The specificity of the modified Seattle, Baltimore, and original Cairo criteria was 87.5%, 99.3%, and 72.2%, respectively. The specificity of the original Cairo criteria was significantly lower than that of the modified Seattle criteria (*p* < 0.001).

### 3.3 Evaluation of the Cairo diagnostic criteria with various cutoff levels

To improve the performance of the Cairo criteria, we next examined these criteria when the cutoff point for the number of events was raised to various levels. When the cutoff point was set to three events, six cases were diagnosed significantly earlier by the Cairo criteria with three events than by the EBMT16 criteria (median, 3.0 days; range, 0–16 days; *p* = 0.036) (Figure 1). While four cases with classical SOS could also be diagnosed earlier by the Cairo criteria with three events than by the modified Seattle criteria, significance was not observed (median 0.0 days; range, 0–5 days; *p* = 0.089). When the cutoff point was set to four events, five cases could be diagnosed earlier by the Cairo criteria with four events than by the EBMT16 criteria, whereas significance was not observed (median, 0.5 days; range 0–9 days; *p* = 0.058).

The sensitivity of the Cairo criteria with cutoff three, four, and five events was 100.0%, 100.0%, and 70.0%, respectively (Figure 3 and Table S3). The specificity of the Cairo criteria with cutoff three, four, and five events was 86.1%, 93.8%, and 100.0%, respectively. There was no significant difference in specificity between the Cairo criteria with three events and the modified Seattle criteria (*p* = 0.789). The specificity of the Cairo criteria with four events was significantly higher than that of the modified Seattle criteria (*p* = 0.027). The ROC curve for the Cairo criteria is shown in Figure 3. Regarding diagnostic precision, the optimal cutoff was identified as four. The diagnostic precision and diagnosed time estimated using each of the criteria are summarized in Table 3.

**TABLE 3:** Summary of the diagnostic precision and diagnosed time estimated using each of the criteria.

	Sensitivity (%), (95% CI)	Specificity (%), (95% CI)	Intervals to EBMT16 diagnosis, days, median (range)
mSeattle	90.0 (55.5–99.7)	87.5 (81.0–92.4)	0.0 (0–9)
Baltimore	100.0 (58.7–100.0)	99.3 (96.2–100.0)	0.0 (0–0)
Cairo 2	100.0 (58.7–100.0)	72.2 (64.2–79.4)	5.0 (0–16)
Cairo 3	100.0 (58.7–100.0)	86.1 (79.4–91.3)	3.0 (0–16)
Cairo 4	100.0 (58.7–100.0)	93.8 (88.5–97.1)	0.5 (0–9)
Cairo 5	70.0 (34.8–93.3)	100.0 (96.2–100.0)	0.0 (−8–2)

Abbreviations: 95% CI, 95% confidence interval; Cairo 2, original Cairo criteria with two or more events; Cairo 3, Cairo criteria with three or more events; Cairo 4, Cairo criteria with four or more events; Cairo 5, Cairo criteria with five or more events; EBMT, European Society for Blood and Marrow Transplantation; mSeattle, modified Seattle criteria.

### 3.4 Association between SOS diagnosis and clinical outcomes

**FIGURE 4:**
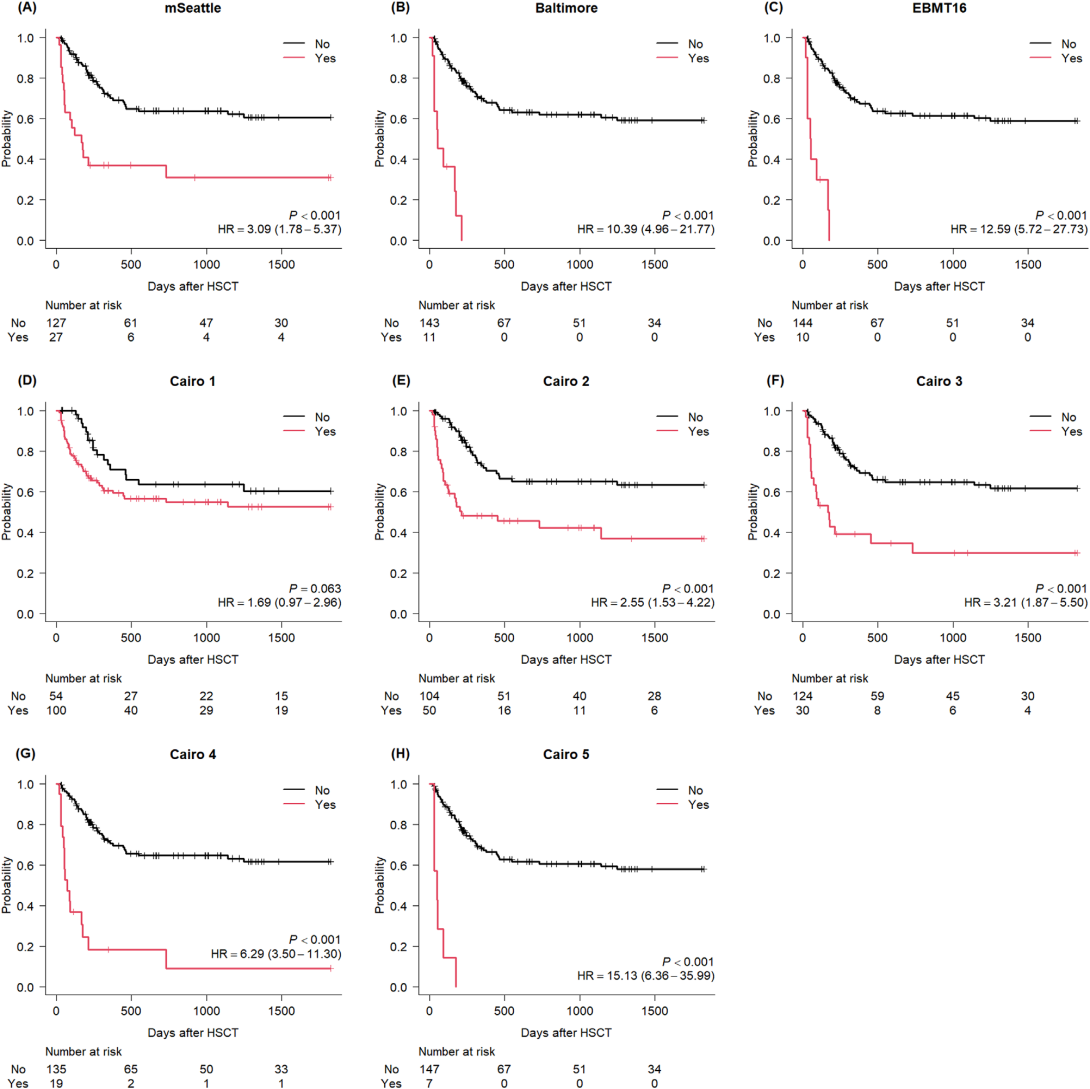
Kaplan–Meier curves of overall survival, grouped by the development of each of the criteria. The hazard ratios and 95% confidence intervals for each criterion are shown. Each proportion of survival at 100 days after HSCT was (A) 59.3% versus 91.8%, (B) 36.4% versus 89.8%, (C) 30.0% versus 89.9%, (D) 78.6% versus 100.0%, (E) 65.4% versus 96.0%, (F) 56.7% versus 93.3%, (G) 36.8% versus 93.1%, and (H) 14.3% versus 89.4%, respectively. Abbreviations: EBMT, European Society for Blood and Marrow Transplantation; HR, hazard ratio; HSCT, hematopoietic stem cell transplantation; mSeattle, modified Seattle criteria.

In all cases, the proportion of survival at 100 days after allo‐HSCT was 85.9% (95% confidence interval [CI], 79.3%–90.6%). The fulfillment of the modified Seattle, Baltimore, or EBMT16 criteria was related to a significant deterioration of overall survival (Figure 4A–C). The proportion of survival at 100 days after allo‐HSCT was 59.3% (95% CI, 38.6%–75.0%), 36.4% (95% CI, 11.2%–62.7%), and 30.0% (95% CI, 7.1%–57.8%) for the modified Seattle, Baltimore, and EBMT16 criteria, respectively. The increased number of cutoff points in the Cairo criteria was related to poor overall survival (Figure 4D–H). The proportion of survival at 100 days after allo‐HSCT for cutoff points one to five were 78.6% (95% CI, 69.1%–85.5%), 65.4% (95% CI, 50.4%–76.9%), 56.7% (95% CI, 37.3%–72.1%), 36.8% (95% CI, 16.5%–57.5%), and 14.3% (95% CI, 0.7%–46.5%), respectively. Overall survival curves grouped by EBMT16 severity grade and Cairo maximum points are shown in Figure S1. The EBMT16 severity was graded for 28 cases who met any established criteria. Both factors tended to be associated with a deterioration in survival.

The proportions of the development of events in the Cairo criteria at the time of fulfilling each criterion are shown in Table 4. In this study, transfusion‐refractory thrombocytopenia was frequently observed in cases with SOS. One case with SOS developed a reversal of portal venous flow detected on Doppler ultrasound. Our study had no instances of “hepatic biopsy consistent with SOS” or “elevated portal venous wedge pressure by direct measurements.”

**TABLE 4:** The proportions of the development of events at the time fulfilling each of the diagnostic criteria.

		*N*	Hyperbilirubinemia	Weight gain	Thrombocytopenia	Hepatomegaly	Pain	Ascites	Doppler
mSeattle (%)	SOS (+)	9	6 (66.7)	9 (100.0)	7 (77.8)	4 (44.4)	5 (55.6)	6 (66.7)	1 (11.1)
	SOS (−)	18	3 (16.7)	17 (94.4)	10 (55.6)	10 (55.6)	8 (44.4)	4 (22.2)	0 (0.0)
Cairo 2 (%)	SOS (+)	10	5 (50.0)	6 (60.0)	9 (90.0)	2 (20.0)	3 (30.0)	3 (30.0)	0 (0.0)
	SOS (−)	40	11 (27.5)	20 (50.0)	34 (85.0)	8 (20.0)	9 (22.5)	5 (12.5)	0 (0.0)
Cairo 3 (%)	SOS (+)	10	7 (70.0)	8 (80.0)	8 (80.0)	4 (40.0)	4 (40.0)	6 (60.0)	1 (10.0)
	SOS (−)	20	8 (40.0)	13 (65.0)	18 (90.0)	10 (50.0)	9 (45.0)	7 (35.0)	0 (0.0)
Cairo 4 (%)	SOS (+)	10	9 (90.0)	9 (90.0)	8 (80.0)	4 (40.0)	5 (50.0)	7 (70.0)	1 (10.0)
	SOS (−)	9	4 (44.4)	8 (88.9)	8 (88.9)	7 (77.8)	4 (44.4)	5 (55.6)	0 (0.0)
EBMT16 (%)		10	10 (100.0)	10 (100.0)	8 (80.0)	4 (40.0)	5 (50.0)	8 (80.0)	1 (10.0)

Abbreviations: Cairo 2, original Cairo criteria with two or more events; Cairo 3, Cairo criteria with three or more events; Cairo 4, Cairo criteria with four or more events; EBMT, European Society for Blood and Marrow Transplantation; mSeattle, modified Seattle criteria.

## 4 DISCUSSION

The original Cairo criteria, defined as the development of two or more events, could diagnose SOS significantly earlier than any other established criteria. As it can lead to the early initiation of SOS treatment, these criteria are considered clinically valuable. The modified Seattle criteria could not diagnose SOS significantly earlier than the EBMT16 criteria could. Defibrotide, known as an effective medication for SOS, has resulted in significant improvement when treating patients with severe SOS [15]. Some studies have demonstrated that a delay in the initiation of defibrotide treatment of over 2 days could significantly decrease the proportion of survival after HSCT [4, 5, 6]. Therefore, early diagnosis is necessary to treat SOS effectively. The original Cairo criteria were superior to the established criteria in terms of the early diagnosis of SOS. However, the specificity of the original Cairo criteria was significantly lower than that of the modified Seattle criteria. Pediatric diagnostic criteria for SOS advocated by the EBMT16, which resemble the Cairo criteria, showed similar results [16, 17, 18]. While a diagnosis with the pediatric criteria was on average 3 days earlier than with the Seattle criteria, the number of patients fulfilling these criteria was extremely high (44.8% of patients undergoing allo‐HSCT) [17]. Similarly, diagnosis with the original Cairo criteria contained a high rate of false positives. As defibrotide may cause fatal side effects, such as hemorrhage [19], the precise diagnosis of SOS is also important. Therefore, we next examined the Cairo criteria when the cutoff point was raised to various levels.

When the cutoff point was set to three events instead of two, the Cairo criteria could also diagnose SOS significantly earlier than the EBMT16 criteria. Diagnostic precision was comparable to that of the modified Seattle criteria. The interval of diagnosis between these criteria and the EBMT16 criteria was approximately 3 days. According to the study on defibrotide mentioned above, a 2‐day delay in SOS diagnosis is associated with worse clinical outcomes [4, 5, 6]. Therefore, this difference is considered clinically important. Consequently, a cutoff of three events would have a greater clinical benefit than the established criteria. When the cutoff point was set to four events, the Cairo criteria could diagnose SOS with precision superior to the modified Seattle criteria. While earlier diagnosis compared to the established criteria was not achieved, in terms of diagnostic precision, this cutoff was also considered clinically useful. When the cutoff point was set to five events or more, these criteria were considered clinically inadequate because of the deterioration in sensitivity. Taken together, the Cairo criteria were considered clinically useful, especially by using three or four event cutoff points. We propose that the development of three events in the Cairo criteria is considered “possible SOS,” and the development of four events is considered “confirmed SOS.” The term “confirmed” derives from the best diagnostic precision in the Cairo criteria. The term “possible” derives from the next best diagnostic precision and the possibility of early diagnosis. The use of these criteria might be associated with early and adequate treatment of SOS and improvements in clinical outcomes.

The Cairo criteria differ from established criteria in its use of “platelet transfusions consistent with refractory thrombocytopenia” as a diagnostic event. Some clinical studies have suggested that refractory thrombocytopenia with excessive platelet transfusions is often observed in SOS [7, 19, 20, 21]. Similar to these studies, refractory thrombocytopenia was frequently observed among the cases with SOS in our study (≥ 80%). However, as thrombocytopenia is a very common finding after allo‐HSCT, the false‐positive rate may be also high. Further investigation into the definition of this event is required. Another event unique to the Cairo criteria, “reversal of portal venous flow detected on Doppler ultrasound,” was observed in only one case with SOS in our cohort. Therefore, this event provided only a minor contribution to the diagnosis. As Cairo et al. mentioned, this result might be based upon the knowledge that this finding tends to be observed in the end stage of SOS [10]. For early diagnosis, the inclusion of this event may require some refinement. Other ultrasound approaches, such as HokUS‐10, seem to be helpful [22, 23].

Clinical literature has suggested that SOS without hyperbilirubinemia is uncommon in adults, unlike in children [7, 16]. However, one study of patients with SOS, including those diagnosed by liver biopsy, showed that SOS without hyperbilirubinemia was observed in approximately 15% of patients [24]. Half of them were diagnosed within 21 days after HSCT. In other words, it is impossible to diagnose SOS in some patients when using the EBMT16 criteria. The fact that some cases in our cohort did not develop hyperbilirubinemia at the fulfillment of the modified Seattle criteria suggested the existence of such patients. The Cairo criteria can diagnose such patients as having SOS properly because hyperbilirubinemia is not mandatory. Similar to previous studies [1, 3], SOS diagnosed using established criteria was associated with an extremely high mortality in our study. Likewise, the development of the Cairo criteria events was associated with high mortality. These results suggest that diagnosis and treatment based on the Cairo criteria are clinically important.

Our study has some limitations. First, because it was a small retrospective trial conducted in a single institution, this study lacked sufficient power. Accordingly, the benefits of the Cairo criteria using a cutoff of three or four events compared with the modified Seattle criteria need further investigation and confirmation. Second, none of the cases with SOS were definitively diagnosed by liver biopsy or autopsy. According to a previous study on patients with SOS undergoing a hepatic biopsy, the sensitivity and specificity of the Baltimore criteria were estimated to be 56% and 92%, respectively [21]. In our study, the EBMT16 criteria were chosen as surrogate reference standards. Therefore, the true diagnostic precision of the Cairo criteria remains unclear. Third, late‐onset SOS was not investigated in this study. The original Cairo criteria do not limit the SOS onset date. In our study, we followed the EBMT16 criteria in which “classical SOS” was defined within 21 days after HSCT. Further studies are needed to evaluate these criteria in late‐onset SOS. Despite these limitations, our study is valuable as the first verification of these criteria.

In conclusion, the Cairo diagnostic criteria could diagnose SOS earlier than the established criteria, with comparable precision. These criteria could be useful for the early and precise diagnosis of SOS, potentially leading to early initiation of treatment and improvement of clinical outcomes. We propose that the development of three events in the present criteria is considered “possible SOS,” and that the development of four events is considered “confirmed SOS.”

## AUTHOR CONTRIBUTIONS

HI and KYak designed the study. HI collected data. HI, KYak, YM, HK, MJ, YH, SM, SN, RS, KK, AK, YS, SK, MI, and HMa provided clinical care to the patients. YM provided regulatory support. HI and KYak performed analyses. HMi supervised the study. HI drafted the manuscript. All authors reviewed, edited, and approved the final manuscript.

## FUNDING INFORMATION

There are no sources of funding to declare for this study.

## CONFLICT OF INTEREST STATEMENT

Kimikazu Yakushijin has received honoraria from Nippon Shinyaku, Jazz Pharmaceuticals, Pfizer, Otsuka Pharmaceutical, and Asahi Kasei Pharma. Akihito Kitao has received honoraria from Nippon Shinyaku and Otsuka Pharmaceutical. Hiroshi Matsuoka has received honoraria from Nippon Shinyaku, Pfizer, and Otsuka Pharmaceutical. Hironobu Minami has received research funding from Nippon Shinyaku, Otsuka Pharmaceutical, and Asahi Kasei Pharma. The remaining authors declare no conflict of interest.

## ETHICS APPROVAL STATEMENT

This study was approved by the Ethics Committee of Kobe University Hospital (No. B220141).

## PATIENT CONSENT STATEMENT

The requirement for informed consent was waived due to the retrospective nature of the study. The patients who participated in this study were offered the opportunity to opt‐out.

## CLINICAL TRIAL REGISTRATION

This study has not been registered for any clinical trial.

## Supporting information

Supporting information

## Data Availability

The data that support the findings of this study are available from the corresponding author upon reasonable request.
