# Supplementary material for: Early diagnosis of sinusoidal obstruction syndrome after hematopoietic stem cell transplantation, with modified diagnostic criteria including refractory thrombocytopenia
Source: EJHaem. 2023 Jun 1;4(3):695–704. doi: 10.1002/jha2.728 (PMC10435725; doi:10.1002/jha2.728)
Supplement: Supplementary file 1 — Supporting information [file JHA2-4-695-s001.docx]

**Supplemental Figure 1. Overall survival grouped by EBMT16 severity grade and maximum Cairo points**


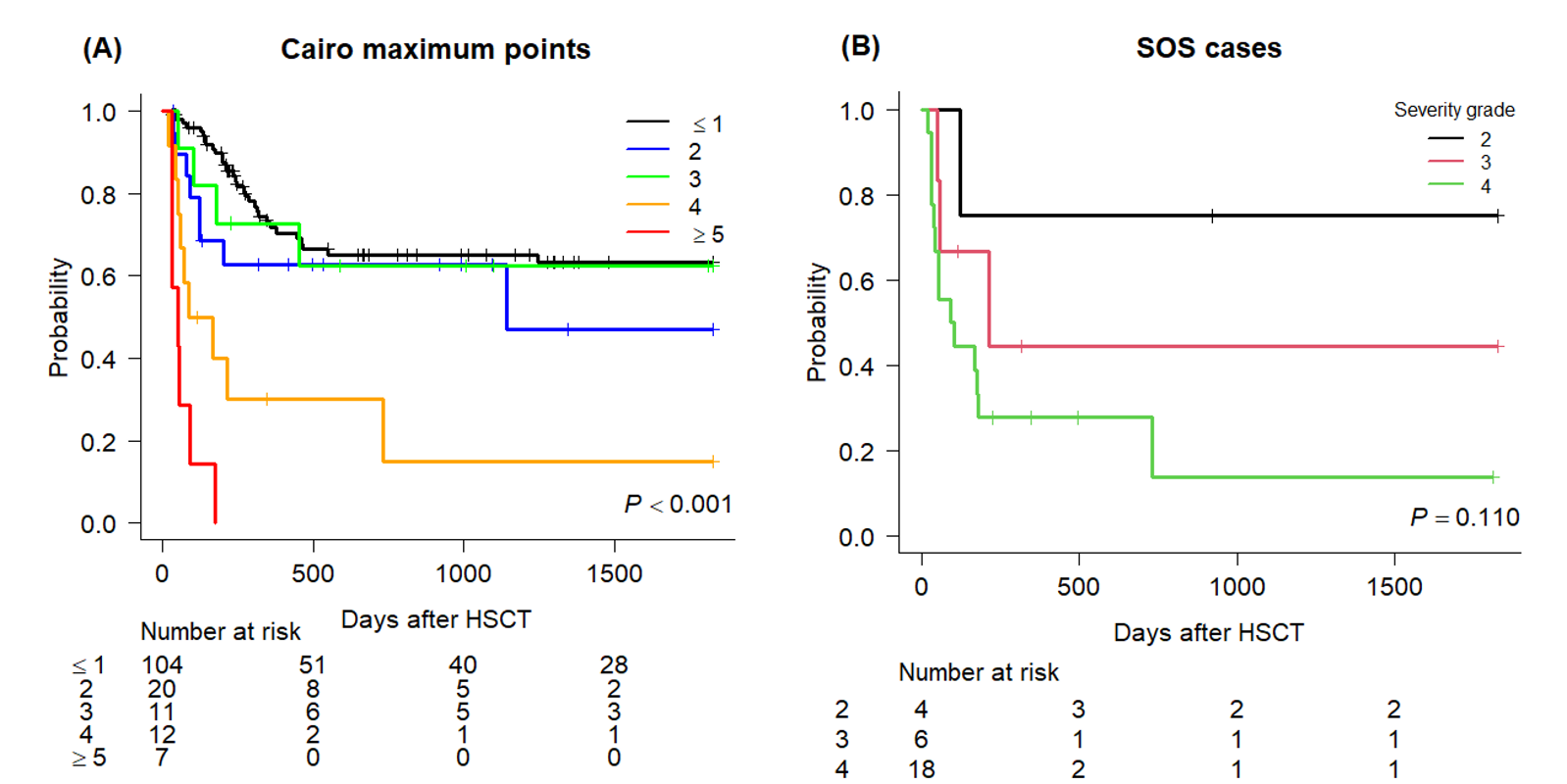


(A) Overall survival grouped by the maximum development of events in the Cairo criteria. (B) Overall survival among 28 cases who developed any established criteria grouped by the severity of SOS.

Abbreviations: HSCT, hematopoietic stem cell transplantation; SOS, sinusoidal obstruction syndrome.

**Supplemental Table 1. Summary of treatment and clinical outcomes for classical SOS cases**

|  | Grade 3 | Grade 4 | Total | *P* value |
| --- | --- | --- | --- | --- |
| N | 2 | 8 | 10 |  |
| SOS remission (%) | 1 (50.0) | 1 (12.5) | 2 (20.0) | 0.84 |
|  |  |  |  |  |
| SOS remission days after HSCT,  median (range) | 35.0 | 32.0 | 33.5 (32.0–35.0) | 0.32 |
|  |  |  |  |  |
| MOF (%) | 1 (50.0) | 7 (87.5) | 8 (80.0) | 0.84 |
|  |  |  |  |  |
| Treatment |  |  |  |  |
| FFP (%) | 0 (0.0) | 5 (62.5) | 5 (50.0) | 0.43 |
|  |  |  |  |  |
| rTM (%) | 0 (0.0) | 4 (50.0) | 4 (40.0) | 0.63 |
|  |  |  |  |  |
| mPSL (%) | 0 (0.0) | 2 (25.0) | 2 (20.0) | 1.00 |
|  |  |  |  |  |
| DF (%) | 2 (100.0) | 1 (12.5) | 3 (30.0) | 0.12 |
|  |  |  |  |  |
| Death (%) | 1 (50.0) | 8 (100.0) | 9 (90.0) | 0.43 |

Abbreviations: SOS, sinusoidal obstruction syndrome; HSCT, hematopoietic stem cell transplantation; MOF, multiple organ failure; FFP, fresh frozen plasma; rTM, recombinant thrombomodulin; mPSL, methylprednisolone; DF, defibrotide.

**Supplemental Table 2. Diagnosed days after HSCT for each of the criteria groups**

|  | N | Diagnosed days after HSCT,  median (range) | Diagnosed days among 10 classical SOS cases,  median (range) |
| --- | --- | --- | --- |
| EBMT16 | 10 | 11.5 (2–20) | – |
| mSeattle | 27 | 11.0 (-6–20) | 10.0 (2–20) |
| Baltimore | 11 | 11.0 (2–20) | 11.5 (2–20) |
| Cairo 2 | 50 | 11.0 (-6–21) | 6.0 (-6–11) |
| Cairo 3 | 30 | 12.5 (1–21) | 6.0 (2–20) |
| Cairo 4 | 19 | 12.0 (2–21) | 9.5 (2–20) |

Abbreviations: HSCT, hematopoietic stem cell transplantation; EBMT, European Society for Blood and Marrow Transplantation; mSeattle, modified Seattle criteria; Cairo 2, original Cairo criteria with two or more events; Cairo 3, Cairo criteria with three or more events; Cairo 4, Cairo criteria with four or more events.

**Supplemental Table 3. Cross tabulation of classical SOS in each of the criteria groups**

|  | Classical SOS (+) | Classical SOS (-) | Total |
| --- | --- | --- | --- |
| N | 10 | 144 | 154 |
| mSeattle (+) | 9 | 18 | 27 (17.5%) |
| mSeattle (-) | 1 | 126 | 127 (82.5%) |
| Baltimore (+) | 10 | 1 | 11 (7.1%) |
| Baltimore (-) | 0 | 143 | 143 (92.9%) |
| Cairo 2 (+) | 10 | 40 | 50 (32.5%) |
| Cairo 2 (-) | 0 | 104 | 104 (67.5%) |
| Cairo 3 (+) | 10 | 20 | 30 (19.5%) |
| Cairo 3 (-) | 0 | 124 | 124 (80.5%) |
| Cairo 4 (+) | 10 | 9 | 19 (12.3%) |
| Cairo 4 (-) | 0 | 135 | 135 (87.7%) |
| Cairo 5 (+) | 7 | 0 | 7 (4.6%) |
| Cairo 5 (-) | 3 | 144 | 147 (95.4%) |

Abbreviations: SOS, sinusoidal obstruction syndrome; mSeattle, modified Seattle criteria; Cairo 2, original Cairo criteria with two or more events; Cairo 3, Cairo criteria with three or more events; Cairo 4, Cairo criteria with four or more events; Cairo 5, Cairo criteria with five or more events.
